# Clinical Patterns and Treatment Outcome in Patients with Melancholic, Atypical and Non-Melancholic Depressions

**DOI:** 10.1371/journal.pone.0048200

**Published:** 2012-10-26

**Authors:** Margalida Gili, Miquel Roca, Silvia Armengol, David Asensio, Javier Garcia-Campayo, Gordon Parker

**Affiliations:** 1 Institut Universitari d'Investigació en Ciències de la Salut (IUNICS), University of Balearic Islands, Palma de Mallorca, Spain; 2 Red de Actividades Preventivas y Promoción de la Salud en Atención Primaria (RediAPP), Barcelona, Spain; 3 Almirall Medical Department, Barcelona, Spain; 4 Department of Psychiatry, Miguel Servet Hospital, University of Zaragoza, Zaragoza, Spain; 5 School of Psychiatry, University of New South Wales, Randwick, New South Wales, Australia; Catholic University of Sacred Heart of Rome, Italy

## Abstract

**Objective:**

To assess sociodemographic, clinical and treatment factors as well as depression outcome in a large representative clinical sample of psychiatric depressive outpatients and to determine if melancholic and atypical depression can be differentiated from residual non-melancholic depressive conditions.

**Subjects/Materials and Method:**

A prospective, naturalistic, multicentre, nationwide epidemiological study of 1455 depressive outpatients was undertaken. Severity of depressive symptoms was assessed by the *Hamilton Depression Rating Scale* (HDRS) and the *Self Rated Inventory of Depressive Symptomatology* (IDS-SR_30_). IDS-SR_30_ defines melancholic and atypical depression according to DSM-IV criteria. Assessments were carried out after 6–8 weeks of antidepressant treatment and after 14–20 weeks of continuation treatment.

**Results:**

Melancholic patients (16.2%) were more severely depressed, had more depressive episodes and shorter episode duration than atypical (24.7%) and non-melancholic patients. Atypical depressive patients showed higher rates of co-morbid anxiety disorders and substance abuse. Melancholic patients showed lower rates of remission.

**Conclusion:**

Our study supports a different clinical pattern and treatment outcome for melancholic and atypical depression subtypes.

## Introduction

The broad heterogeneity of clinical depression has long encouraged research seeking to identify depressive subtypes that show causal, and even more importantly, treatment specificity [1], [2]. As yet inconsistencies in research findings have failed to convincingly demonstrate absolute depressive sub-types, contributing to the default model of differentiating depressive states dimensionally. The historical controversy about the nature, definition and classification of atypical and melancholic subtypes [3]–[5] in particular has gained strength in preparation of the DSM-5 manual [6], [7]. Differing options for future DSM depressive categories include weighting depressive sub-types, as against providing specifiers for major depressive episodes or distinct qualitative affective disorders [1], [4], [5], [8]–[12].

According to previous studies, melancholic depression affects about 25–30% of depressive populations [13], [14] and is clinically characterized by distinct quality of mood, non-reactivity of mood to circumstances, anhedonia, psychomotor disturbance, cognitive impairment and symptoms of vegetative dysfunction such as terminal insomnia, diurnal mood variation with worsening in the morning and weight loss [7], [15]. From a biological perspective, hypercortisolemia, neuroimaging features and disturbances in sleep architecture have been associated with melancholia [4], [10]. Melancholic patients are held to respond better to Electroconvulsive therapy (ECT) and to certain pharmacological approaches such as tricylic antidepressants (TCA) rather than to selective serotonin reuptake inhibitors (SSRIs) [16]. Compared to non-melancholic depression, melancholia rarely responds to placebos, psychotherapies or social interventions [17]. Depressive patients with melancholic features have worse outcomes and reduced probability of remission from major depressive disorder compared to those with non-melancholic depression [18]. Some authors have therefore argued that melancholia is a disease entity on the basis of its psychopathology, biology and differential response to treatment [12] and have proposed new diagnostic criteria [4].

By contrast, atypical depression (initially contrasted with the so-called ‘typical’ endogenous or melancholic depressive condition) is characterized by significant mood reactivity, severe fatigue, anxiety, hypersomnia, increased appetite and a personality style of rejection sensitivity. Selective response to monoamine oxidase inhibitors (MAOIs), polysomnographic changes and endocrine features have been interpreted by some authors as also positioning atypical depression as a distinct entity [19] while others have argued for the primacy of a personality style rejection sensitivity and response to salient stressors [20], [21]. This condition appears common, with studies indicating rates ranging from 20% to 35% of depressed patients [21], [22], while it appears to have an earlier age of onset and a more chronic course of illness than melancholia [23].

While such studies prioritize these depressive subtypes as the most promising categorical candidates in future DSM-5 classificatory model, the available evidence is largely limited to clinical trial studies with narrow inclusion criteria and very stringent treatment conditions. The naturalistic design of the present study provides an opportunity to assess sociodemographic, clinical and treatment factors as well as depression outcome in a large representative clinical sample of psychiatric depressive outpatients, to determine if melancholic and atypical depression can be positioned as distinctive clinical entities.

## Methods

### Study design and population

The main objectives and details of the RESIST study have been described previously [24]. Briefly, the RESIST is a large prospective naturalistic multicentre study conducted in regional outpatient Spanish settings. Four hundred psychiatrists proportionally distributed by regions within Spain's 17 regional communities were selected to participate, and each asked to recruit five outpatients. Inclusion in the study required participants to sign a written informed consent, to be over 18 years of age, to meet Major Depression diagnoses according to DSM-IV criteria, and to have had 6–8 weeks of antidepressant drug treatment.

Recruitment took place under naturalistic clinical conditions in outpatient settings. Data were collected during two routine visits after obtaining written consent, and with the first assessment occurring after at least six weeks of antidepressant therapy.

A total of 374 (86%) psychiatrists accepted the invitation to contribute, and 1870 patients were initially recruited. Of those, 140 patients were excluded from the current analyses as they were in remission at first assessment, 275 were excluded due to: change of treatment (n = 171, 9.1%), patients not having a second assessment (n = 68, 3.6%) incomplete or missing data (n = 36, 1.9%), leaving 1595 patients in the provisional sample. Data collection took place from February to June 2009 after receiving the approval of the Teknon Medical Center ethical committee (Barcelona, Spain). Thus, our analyses were undertaken on a sample of 1455 patients.

### Measures

#### Sociodemographic and clinical characteristics

Data collected at the first visit included sociodemographic characteristics (age, gender, current occupation, marital status, education, living status and environment), history and clinical features of the depressive disorder (age at onset, first or recurrent episode, number of previous episodes, length of current episode), DSM-IV-TR comorbid psychiatric diagnoses and comorbid medical diseases.

#### Severity and improvement assessment

The severity of depressive symptoms was assessed by the *Hamilton Depression Rating Scale* (HDRS) and the *Self Rated Inventory of Depressive Symptomatology* (IDS-SR_30_). The HDRS_21_ includes 21 items, each rated on a 0–2 or 0–4 scale by the clinician, with a range for the total score from 0 (without depressive symptoms) to 66 (severe depressive symptoms).

The IDS-SR_30_ assesses 30 symptoms obtaining a total score with a range from 0 (without depressive symptoms) to 84 (severe depressive symptoms). At each assessment the HDRS_21_ and IDS-SR_30_ measures were administered. The IDS-SR_30_ to assess all core criterion diagnostic depressive symptoms as well as DSM-IV atypical and melancholic symptom features [25], [26].

### Definition of melancholic and atypical depression

For the purpose of this study, melancholic and atypical depression were defined using algorithms from selected items of IDS-SR_30_ developed by the STAR*D research group [13], [22], and with such definitions corresponding to DSM-IV criteria. According to those specific definitions for assigned melancholic depression, patients were required to score 2 or 3 on the IDS-SR_30_ anhedonia and non-reactive mood items and score positively on at least 3 of the following criteria: *distinct* quality of mood, diurnal mood variation with worsening symptoms in the morning, psychomotor retardation, psychomotor agitation, appetite or weight decrease, early morning awakening and self-outlook. For assigned atypical depression, patients were required to score 0, 1 or 2 for mood reactivity and affirm at least two of the following items: 2 or 3 for leaden paralysis, 2 or 3 for weight gain or increased appetite, 2 or 3 for hypersomnia, and 3 for interpersonal sensitivity.

### Antidepressant treatment characteristics

Treatment modality was determined by the clinician's individual decision. Any drug type, dose, regimen of antidepressant or concomitant medication was allowable and entirely at the discretion of the psychiatrist. Change of treatment for any reason resulted in exclusion from the study. Description of antidepressant treatment was collected at the second visit. For the present analysis antidepressant types were divided into three categories: Selective Serotonin Reuptake Inhibitors (SSRIs), Selective Serotonin- Norepinephrine Reuptake Inhibitors (SNRIs) and Trycyclic Antidepressants (TCAs). Antidepressant regimes were classified as monotherapy (using only one antidepressant during the study period) and combination therapy (using more than one antidepressant during the study period). Concomitant medication was categorized as additional use of antipsychotic, mood stabilizer and anxiolytic/hypnotic (benzodiazepine) medication.

### Definition of treatment outcomes

HDRS_21_ remission was defined as having a HDRS_21_ score ≤7, and IDS-SR_30_ remission was defined as having an IDS-SR_30_ score ≤14 after 16–20 weeks of antidepressant treatment.

### Statistical analyses

T-tests compared differences between groups for quantitative variables. Chi-square tests and unadjusted odds ratios (ORs) were calculated to explore differences between patient groups for qualitative variables. In order to assess the relative strength of each variable, we ran separate binary logistic regression analyses. Each of these regressions was re-run controlling for age, gender and severity of depression as they were significantly different. These analyses examined for group differences and to quantify the magnitude of effects, being reported as *adjusted* ORs with 95% C.I. These control factors (age, gender and severity) were chosen to enhance our comparison procedures and to ensure that group differences were not likely due to differences on the demographic and clinical factors. Statistical analyses were conducted using SPSS for Windows Version 19.0. A probability level of 0.05 was considered statistically significant.

## Results

### Sociodemographic variables

Final analyses were undertaken on 1455 subjects. Study criteria assigned 237 (16.2%) as having melancholic depression and 360 (24.7%) as having atypical depression. Sociodemographic characteristics associated with melancholic and atypical depression (after adjusting for severity of depression) are summarized in Table 1. Depressive subtypes did not differ significantly across sociodemographic variables except for gender and age. Melancholic patients were slightly older than non-melancholic and atypical depressive patients. Males were over-represented with a melancholic diagnosis whereas females had higher rates of atypical depression.

**Table 1:** Sociodemographic characteristics associated to melancholic and atypical depression.

Variable		Melancholic (n = 237)		Non Melancholic (n = 1218)		Atypical (n = 360)		Non Atypical (n = 1095)		Melancholic vs Non Melancholic	Atypical vs non Atypical	Melancholic vs Atypical
		n	Mean (SD) or %	n	Mean (SD)or %	n	Mean (SD) or %	n	Mean (SD) or %	t, p or OR (95% CI) #	t, p or OR (95% CI) #	t, p or OR (95% CI) #
**Age-yr**		237	48.7 (12.3)	1218	47.5 (13.1)	360	47.2 (12.4)	1095	47.8 (13.2)	t = −1.32, p = 0.018	t = 0.78, p = 0.430	t = 1.44, p = 0.15
**Gender**	**Male**	96	19.1	406	80.9	107	21.3	395	78.7	−	−	−
	**Female**	141	14.8	812	85.2	253	26.5	700	73.5	OR = 1.5 (1.07−2.1)*	OR = 0.63 (0.47−0.85)**	OR = 0.53 (0.36−0.79)**
**Employment**	**Employed**	99	14.7	569	85.3	165	24.5	504	75.5	−	−	−
	**Unemployed**	35	18.4	154	81.6	46	25.1	140	74.9	OR = 0.72 (0.46−1.13)	OR = 0.9 (0.6−1.35)	OR = 1.21 (0.72−2.03)
	**Housework**	61	17.1	305	82.9	91	25.3	274	74.7	OR = 1.08 (0.62−1.87)	OR = 0.9 (0.55−1.48)	OR = 0.93 (0.48−1.81)
	**Retired**	42	20.2	190	79.8	58	25	177	75	OR = 1.07 (0.66−1.75)	OR = 0.8 (0.52−1.23)	OR = 0.83 (0.46−1.46)
**Marital status**	**Never married**	34	11.9	252	88.1	67	23.4	219	76.6	−	−	−
	**Married**	152	17.2	734	82.8	225	25.4	661	74.6	OR = 0.72 (0.42−1.26)	OR = 1.02 (0.65−1.62)	OR = 1.32 (0.7−2.49)
	**Widowed**	20	19.6	82	80.4	20	19.6	82	80.4	OR = 0.86 (0.52−1.42)	OR = 1.27 (0.82−1.97)	OR = 1.25 (0.68−2.29)
	**Divorced**	31	17.1	150	82.9	48	26.5	133	73.5	OR = 1.09 (0.55−2.19)	OR = 0.71 (0.37−1.34)	OR = 0.63 (0.26−1.48)
**Education**	**Incomplete primary**	46	17	225	83	67	24.7	204	75.3	−	−	−
	**Complete primary**	81	16.7	405	83.3	119	24.5	367	75.5	OR = 0.84 (0.47−1.49)	OR = 0.78 (0.48−1.26)	OR = 0.98 (0.51−1.87)
	**Secondary**	77	16.5	391	83.5	107	22.9	361	77.1	OR = 0.89 (0.55−1.45)	OR = 0.78 (0.53−1.16)	OR = 0.91 (0.52−1.57)
	**University**	33	14.3	197	85.7	67	29.1	163	70.9	OR = 1.06 (0.67−1.67)	OR = 0.72 (0.50−1.04)	OR = 0.74 (0.44−1.26)
**Lives**	**Alone**	39	15.1	220	83.4	70	27	189	73	−	−	−
	**Accompanied**	198	16.6	998	81.9	290	24.2	906	75.8	OR = 0.84 (0.52−1.36)	OR = 1.36 (0.91−2.04)	OR = 1.58 (0.89−2.8)
**Environment**	**Rural**	68	17	332	83	89	22.3	311	77.8	−	−	−
	**Urban**	169	16	886	84	271	25.7	784	74.3	OR = 1.01 (0.72−1.4)	OR = 0.85 (0.63−1.14)	OR = 0.89 (0.59−1.33)

# Adjusted for IDS-SR_30_ depression severity *p<0.05**p<0.01

### Clinical variables

Melancholic patients were more severely depressed than non- melancholic patients after 6-8 weeks of Antidepressant Treatment (ADT) (mean 53.8, SD 9.1 vs 34.1, SD 11.2 on the IDS-SR_30_ measure and 26.7, SD 6.1 vs 16.9, SD 6.9 on the HDRS_21_ measure) and after 16–20 weeks (mean 19.1, SD 12.8 vs 15.6, SD 10.5–IDS-SR_30_, and 8.7, SD 6.4 vs 7.1, SD 5.0, HDRS_21_). Further, melancholic patients were more severely depressed than atypical patients after 6–8 weeks of treatment (mean 53.8, SD 9.1 vs 43.2, SD 10.0 in IDS-SR_30_ and 26.7, SD 6.1 vs 20.5, SD 7.0 on HDRS_21_) but did not differ in severity after 16–20 weeks. Also there were severity differences between atypical and non-atypical subjects after 6-8 weeks (mean 43.2, SD 10.0 vs 35.7, SD 13.4–IDS-SR_30_, and 20.5, SD 7.0 vs 17.9, SD 7.7, HDRS_21_ and after 16–20 weeks of treatment (mean 18.9, SD 11.9 vs 15.3, SD 10.5–IDS-SR_30_, and, 8.41, SD 5.5 vs 7.04–SD 5.2–HDRS_21_).

There were no statistical differences in age at depression onset but melancholic patients had more depressive episodes than the other depressed subjects (4.3 vs 3.6 in atypical and 3.6 in non-melancholic) and with shorter episode duration (11.6 weeks vs 14.4 in non-melancholic vs 14.4 in atypical).

Atypical depressive patients had higher rates of comorbid anxiety disorders (43.9% vs 34.2% in melancholic patients; OR = 1.35, CI = 1.06–1.7) and higher rates of co-morbid substance abuse (13.3% vs 8.4% in melancholic patients; OR = 0.52, CI = 0.3–0.79). (Table 2)

**Table 2:** Clinical characteristics associated to melancholic and atypical depression.

Variable		Melancholic (n = 237)		Non Melancholic (n = 1218)		Atypical (n = 360)		Non Atypical (n = 1095)		Melancholic vs Non Melancholic	Atypical vs non Atypical	Melancholic vs Atypical
		n	Mean (SD) or %	n	Mean (SD) or %	n	Mean (SD) or %	n	Mean (SD) or %	t, p or OR (95% CI) #	t, p or OR (95% CI) #	t, p or OR (95% CI) #
**Age at onset of first MDE-years**		237	39.9 (13.6)	1218	40.5 (13.4)	360	40.2 (12.9)	1095	40.5 (13.7)	t = 0.67, p = 0.49	t = 0.45, p = 0.64	t = −0.23, p = 0.81
**Episode**	**First episode**	118	49.8	654	53.7	182	50.6	590	53.9	−	−	−
	**Recurrent episode**	119	50.2	564	46.3	178	49.4	505	46.1	OR = 0.96 (0.71−1.29)	OR = 0. 95 (0.74−1.23)	OR = 1.01 (0.72−1.42)
**Number of previous episodes**		119	4.35 (3.4)	564	3.67 (2.8)	178	3.61 (2.8)	505	3.85 (2.9)	t = 2.3, p = 0.02	t = 0.94, p = 0.34	t = −2.01, p = 0.04
**Length of current episode**−**weeks**		237	11.6 (7.1)	1218	14.4 (9.6)	360	14.4 (9.7)	1095	13.8(9.1)	t = 5.02, p<0.000	t = −1.14, p = 0.25	t = −3.76, p<0.000
**Severity ADT treatment**	**HDRS_21_ after 6–8 weeks of**	237	26.7 (6.1)	1218	16.9 (6.9)	360	20.5 (7.0)	1095	17.93 (7.7)	t = −20.2, p<0.000	t = 5.55, p<0.000	t = 11.08, p<0.000
	**HDRS_21_ after 16–20 weeks of ADT treatment**	237	8.7 (6.4)	1218	7.12 (5.0)	360	8.41 (5.5)	1095	7.04 (5.2)	t = −4.18, p<0.000	t = −4.24, p<0.000	t = 0.58, p = 0.55
	**IDS-SR_30_ after 6–8 weeks of ADT treatment**	237	53.8 (9.1)	1218	34.1 (11.2)	360	43.27 (10.0)	1095	35.4 (13.4)	t = −25.2, p<0.000	t = −10.1, p<0.000	t = 12.9, p<0.000
	**IDS-SR_30_ after 16–20 weeks of ADT treatment**	237	19.1 (12.8)	1218	15.6 (10.5)	360	18.96 (11.9)	1095	15.3 (10.5)	t = −4.57, p<0.000	t = −5.5, p<0.000	t = 0.56, p = 0.57
**Comorbid Anxiety disorder**	**Yes**	81	34.2	477	39.2	158	43.9	400	36.5	−	−	−
	**No**	156	65.8	741	60.8	202	56.1	695	63.5	OR = 1.25 (0.93−1.7)	OR = 1.35 (1.06−1.7)*	OR = 0.68 (0.48−0.96)*
**Comorbid Substance abuse disorder**	**Yes**	20	8.4	117	9.6	48	13.3	89	8.1	−	−	−
	**No**	217	91.6	1101	90.4	312	86.7	1006	91.1	OR = 1.25 (0.74−2.1)	OR = 0.52 (0.3−0.79)**	OR = 0.62 (0.35−1.09)*
**Comorbid Medical disease**	**Yes**	115	48.5	536	44	168	46.7	483	44.1	−	−	−
	**No**	122	51.5	682	56	192	53.3	612	55.9	OR = 0.99 (0.73−1.3)	OR = 0.94 (0.72−1.23)	OR = 1.07 (0.46−1.49)

# Adjusted for gender, age and IDS-SR_30_ severity of depression *p<0.05 **p<0.01

### Treatment outcome

Measures of remission and their association to melancholic and atypical depression are compared in Table 3. The remission rates (as quantified by the HDRS_21_ and IDS-SR_30_) were significantly lower in melancholic patients compared with non-melancholic and atypical patients after adjustment for age, gender and severity at 6–8 weeks. Melancholic subjects showed a lower probability of remission (OR = 0.63, CI = 0.5–0.81–IDS-SR_30_ and OR = 0.75, CI = 0.59–0.96–HDRS_21_) than atypical subjects.

**Table 3:** Remission associated to depressive subtypes.

Variable		Melancholic (n = 237)		Non Melancholic (n = 1218)		Atypical (n = 360)		Non Atypical (n = 1095)		Melancholic vs Non Melancholic	Atypical vs non Atypical	Melancholic vs Atypical
		n	Mean (SD) or %	n	Mean (SD) or %	n	Mean (SD) or %	n	Mean (SD) or %	OR (95% CI) #	OR (95% CI) #	OR (95% CI) #
**IDS-SR_30_ remission**	**Yes**	91	38.4	638	52.4	150	41.7	579	52.9	−	−	−
	**No**	146	61.6	580	47.6	210	58.3	516	47.1	0.56(0.42−0.75)**	0.63(0.5−0.81)**	1.14(0.8−1.6)**
**HDRS_21_ remission**	**Yes**	118	49.5	733	60.2	192	53.3	659	60.2	−	−	−
	**No**	119	50.2	485	39.8	168	46.7	436	39.8	0.65(0.49−0.86)**	0.75(0.59−0.96)*	1.15(0.83−1.6)**

# Adjusted for gender, age and IDS-SR_30_ severity of depression

* p<0.05

** p<0.01

### Treatment-related variables

Treatment-related characteristics of the sample and differences by depressive subtypes are summarized in Table 4. Melancholic subjects received higher rates of SNRI medication than non-melancholic subjects (OR = 1.5, CI = 1.1–2.0) and lower rates of SSRI medication than non-melancholic (OR = 0.4, CI = 0.2–0.6) and atypical patients (OR = 2.0, CI = 1.3–3.2). Melancholic patients also had higher rates of receiving concomitant antipsychotic medication (OR = 2.7, CI = 1.7–4.4).

**Table 4:** Treatment related characteristics associated to depressive subtypes.

Variable		Melancholic (n = 237)		Non Melancholic (n = 1218)		Atypical (n = 360)		Non Atypical (n = 1095)		Melancholic vs Non Melancholic	Atypical vs non Atypical	Melancholic vs Atypical
		n	Mean (SD) or %	n	Mean (SD) or %	n	Mean (SD) or %	n	Mean (SD) or %	OR (95% CI) #	OR (95% CI) #	OR (95% CI) #
**Antidepressant regimen**	**Monotherapy**	174	73.4	939	77.1	267	74.2	846	77.3	0.8 (0.5−1.1)	0.84 (0.6−1.1)	1 (0.7−1.5)
	**Combination**	63	26.6	279	22.9	93	25.8	249	22.7	−	−	−
**Antidepressant type**	**SSRI**	31	13.1	328	26.9	85	23.7	274	25	0.4 (0.2−0.6)**	0.9 (0.7−1.2)	2 (1.3−3.2)**
	**SNRI**	167	70.5	744	61.1	227	63.1	684	62.5	1.5 (1.1−2)**	1 (0.8−1.3)	0.7 (0.5−1)
	**TCA**	23	9.7	95	7.8	33	9.2	85	7.8	1.2 (0.7−2)	1.1 (0.7−1.8)	0.9 (0.5−1.6)
**Concomitant medication**	**Antipsychotics**	28	11.8	56	4.6	22	6.1	62	5.7	2.7 (1.7−4.4)**	1 (0.6−1.7)	0.4 (0.2−0.8)**
	**Mood stabilizers**	7	3	29	2.4	10	2.8	26	2.4	1.2 (0.5−2.8)	1.1 (0.5−2.4)	0.9 (0.5−1.7)
	**Benzodiazepines**	127	53.6	649	53.3	198	55	578	52.8	1 (0.7−1.3)	1.1 (0.8−1.3)	1 (0.7−1.4)

# Adjusted for gender, age and IDS-SR_30_ severity of depression

* p<0.05

** p<0.01

## Discussion

In our sample, 16.2% of the patients exhibited melancholic features and 24.7% atypical features of depression. Our study provides further empirical evidence in support of a different clinical profile and treatment outcome in melancholic and atypical depressive patients in comparison to those with non-melancholic depression, thus arguing for their positioning as qualitatively distinct from other forms of depression. Comparing both groups, melancholic patients were predominantly male, older, had higher depression severity scores, lower remission rates, more previous depressive episodes, while they were treated with SNRI and antipsychotics drugs more frequently. Patients with atypical depression were more likely to be female, younger, to have less severe depression, fewer episodes, longer duration of episodes, and higher comorbidity involving anxiety and substance abuse disorders. Both groups show no differences in other sociodemographic variables or in age of onset of their condition. Our study also supports the validity of melancholic and atypical depression as clinical subtypes differing from each other and from non-melancholic depressive patients.

The finding than men were more likely to be diagnosed as melancholic and women more likely to be diagnosed as having an atypical depression is consistent with published studies [13], [21], [22], [27]. Older age in melancholic patients has also been described in previous works [28], [29]. As no sociodemographic variables except gender and age showed differences between the studied groups, results support the hypothesis that psychosocial determinants have a limited role as contributing to these depressive subtypes. As overviewed in the Introduction, biological factors are likely to play a more relevant role in the development of melancholic or atypical clinical syndromes.

In our sample melancholic subjects were more likely to have briefer and more severe current index depressive episodes. In contrast to most [22], [30] but not all previous studies [31], [32], we found no support for older age at initial onset in melancholic patients or an earlier age of onset and a more chronic course for atypical depression [33]. Melancholia has been associated with severity and with a shorter duration of the index episode in the STAR*-D cohort and a slightly lower age at the time of study entry [13]. In fact, a surprising finding of our sample was a significant higher number of previous episodes in melancholic patients compared with non-melancholic and atypical depressive patients. Despite the clinical and co-morbidity differences between first and recurrent affective episodes [24], [34], there are no long-term outcome studies on depressive features and recurrence or chronicity.

In our data, atypical depression was associated with several differing clinical characteristics when compared with melancholic patients: less depression severity, fewer episodes, longer duration of episodes and higher comorbidity with anxiety and substance abuse disorders. It has been reported that patients with atypical depression have an earlier age of onset and a more chronic course of illness compared with melancholic ones [34]. In the STAR*-D cohort, participants with atypical features were more likely to be younger at depression onset, to have a longer index episode, a positive history of suicide, lower remission rates and anxious features or chronic depression [35].

Regarding anxiety and comorbid substance abuse disorder, while our findings are consistent with previous studies [20], [36], [37], it should be noted that some symptoms that are part of the definition of atypical depression used for this study (i.e. leaden paralysis and interpersonal rejection) are associated with anxiety itself and may have confounded results related to anxiety comorbid disorders. Links between atypical depression and comorbid anxiety deserve further investigation.

An important finding related to treatment outcome was the lower remission rate among individuals with melancholic and atypical depression. The naturalistic design of the study gives a special significance to this result as the majority of previous evidence comes from clinical trials comparing few antidepressant options and restricted inclusion criteria. The available data on melancholic depression favors tricyclic than narrow-action antidepressants [7], [38], [39]. A meta-analysis of 38 double-blind studies concluded that the reversible MAOI moclobemide have higher response rates in depressed patients with melancholic features[40]. While SSRI have shown efficacy compared to placebo in some studies [41], they appear less effective compared with the SNRI venlafaxine [42]. However, the majority of those studies considered response but not remission as a primary endpoint. When remission is considered and compared, remission rates in melancholic depression with TCA were significantly better than with SSRI [16]. In the STAR*-D cohort, melancholic depression (23.5% of 2,875 depressive patients included) was associated with a significant reduced rate of remission with citalopram, an SSRI. According to the authors of this STAR*-D report, this result could be attributed to the overlap between melancholic symptoms and core depressive symptoms rated by the assessment instruments [17]. In atypical depression, the treatment data are quite controversial. MAOIs were reported as superior to TCA s in one study [43]. However, fluoxetine was superior to nortryptiline in another study [44], while response and remission rates were similar between sertraline, fluoxetine and moclobemide in depressed patients with atypical features [45], [46].

Our study was not a comparative study of pre-selected antidepressants drugs, and it was intriguing that in such a ‘real world’ clinical setting we found that melancholic patients were treated more frequently with SNRI and antipsychotic medications. Our group of melancholic patients exhibited greater severity and at the same time lowers remission rates and more previous episodes. The combination of antidepressants plus antipsychotics drugs is currently one of the most evident strategies for resistant depression, and would appear to be preferentially provided by our clinicians to those with a melancholic depression.

A number of study limitations are offered in interpreting the results. First, the use of derived Hamilton and IDS-SR_30_ item scores to capture melancholic and atypical patients risks being somewhat arbitrary. As melancholia requires some symptoms to be present, melancholic patients tend to score higher on severity scales [47], [48], and it therefore remains unclear as to whether assigned melancholic patients therefore differed by type or by severity. It is difficult to differentiate between antidepressant drug response and clinical characteristics of the disorder. Second, baseline scores previous to pharmacological treatment were not assessed. Finally, TCAs and MAOIs are not currently used in clinical practice in our country while ECT was not prescribed by clinicians in our outpatient sample despite the published data on the efficacy of this treatment [49], [50] and psychotherapy was not considered in data analyses. For that reason, conclusions on treatment differences between the groups needs further research clarification.

The main strengths of this study were its naturalistic design and large sample size, allowing differences between potential depressive sub-types to be pursued with some confidence.

In conclusion, our findings suggest important clinical pattern and remission differences in depressive outpatients with melancholic and atypical features. The clinical significance of these results is that it might be important to assess melancholic or atypical features in depressive patients prior to commencing treatment as such diagnostic decisions may contribute beneficially to treatment selection.
